# Performance of the Dexcom G6 Continuous Glucose Monitoring System in Pregnant Women with Diabetes

**DOI:** 10.1089/dia.2020.0085

**Published:** 2020-12-07

**Authors:** Kristin Castorino, Sarit Polsky, Grenye O'Malley, Camilla Levister, Kristen Nelson, Christian Farfan, Scott Brackett, Sarah Puhr, Carol J. Levy

**Affiliations:** ^1^ Sansum Diabetes Research Institute, Santa Barbara, California, USA.; ^2^ Barbara Davis Center for Diabetes, University of Colorado Anschutz Medical Campus, Aurora, Colorado, USA.; ^3^ Department of Medicine, Icahn School of Medicine at Mount Sinai, New York, New York, USA.; ^4^ Dexcom, Inc., San Diego, California, USA.

**Keywords:** Continuous glucose monitoring, Accuracy, Performance, Safety, Pregnancy, Gestational diabetes

## Abstract

***Background:*** The aim of this study was to determine the performance of the Dexcom G6 continuous glucose monitoring (CGM) system across three sensor wear sites in pregnant women with diabetes in the second or third trimesters.

***Methods:*** Participants with type 1 (T1D), type 2 (T2D), or gestational (GDM) diabetes mellitus were enrolled at three sites. Each wore two G6 sensors on the abdomen, upper buttock, and/or posterior upper arm for 10 days and underwent a 6-h clinic session between days 3 and 7 of sensor wear, during which YSI reference blood glucose values were obtained every 30 min. No intentional glucose manipulations were performed. Accuracy metrics included the proportion of CGM values that were within ±20% of paired reference values >100 mg/dL or ±20 mg/dL of YSI values ≤100 mg/dL (hereafter referred to as %20/20), as well as the analogous %15/15, %30/30, and %40/40. The mean absolute relative difference (MARD) between CGM-YSI pairs was also calculated.

***Results:*** Thirty-two participants with T1D (*n* = 20), T2D (*n* = 3), or GDM (*n* = 9) were enrolled: 19 were in the second trimester and 13 were in the third trimester of pregnancy. Compared with the reference, 92.5% of CGM values were within ±20%/20 mg/dL. The overall MARD and that of sensors worn on the abdomen, upper buttock, and posterior upper arm was 10.3%, 11.5%, 11.2%, and 8.7%, respectively. There were no device-related adverse events. Skin reactions at the insertion sites were absent or minor.

***Conclusions:*** The Dexcom G6 CGM system is accurate and safe in pregnant women with diabetes.

## Introduction

Pregnancies complicated by diabetes, whether pregestational type 1 (T1D), pregestational type 2 (T2D), or gestational (GDM) diabetes mellitus, have higher rates of adverse maternal and neonatal outcomes. These include spontaneous abortion, stillbirth, large-for-gestational-age (LGA) infants, and neonatal hypoglycemia.^1–3^ Adverse obstetric outcomes during a pregnancy complicated by diabetes can be mitigated by normalization or near normalization of maternal glucose concentrations^4,5^; consequently, more stringent glucose targets for pregnant women with diabetes are recommended.^6–8^ However, efforts to improve maternal glycemic control are complicated by a dramatic increase in hypoglycemia risk during pregnancy as severe hypoglycemia is three to five times as frequent in early pregnancy compared with pre-pregnancy.^9–14^ Hypoglycemia risk is related to the very tight glycemic goals and to the fluctuations in insulin sensitivity during pregnancy,^15–17^ together necessitating frequent insulin titration and consistent glucose monitoring.

Self-monitoring of blood glucose (SMBG) is commonly used during pregnancies complicated by diabetes. However, SMBG testing is painful, volitional, and obtrusive. Many women with diabetes do not test at the recommended frequency, which may be associated with poor pregnancy outcomes^18^; SMBG results often fail to detect trends or short-lived glycemic excursions.^19–21^ Continuous glucose monitoring (CGM) addresses many of these shortcomings, as current CGM systems can be used for insulin dosing decisions, do not require routine calibration, can display data locally or remotely, and can automatically alert users (or their remote followers) to abnormal glucose values or trends.

These improvements have contributed to increasing numbers of patients who use CGM, and higher proportions of time in which the devices are used.^22^ In particular, self-reported rates of CGM usage among pregnant or recently pregnant women with diabetes have rapidly increased over the past decade. In 2010–2013, 36% of the 214 women who participated in the T1D Exchange Clinic Network and Registry in the United States and were pregnant at enrollment or at year-1 follow-up (recently pregnant) reported using CGM.^23^ The CGM use during pregnancy was significantly higher in recently pregnant women than in ever-pregnant women (34% vs. 15%, *P* = 0.006). Recently, pregnant women also had the lowest A1C levels of all the cohorts (6.5% recently pregnant vs. 7.8% ever pregnant vs. 8.0% never pregnant, *P* < 0.001).^23^ By 2016–2018, 70% of pregnant women participating in the T1D Exchange reported using CGM during pregnancy.^22^

The CONCEPTT study^24,25^ revealed the safety and clinical benefits of CGM usage in pregnant women, demonstrating that CGM use in mothers with T1D in the United Kingdom is cost-effective and associated with significant improvements in maternal time in the target glucose range (63–140 mg/dL), significantly lower maternal glucose at 24-weeks' and 34-weeks' gestation, and clinically relevant reductions in LGA infants, neonatal hypoglycemia requiring intravenous dextrose, and neonatal intensive care unit admissions compared with SMBG alone.^24–29^

In recognition of the superiority of modern-generation CGM systems over traditional glucose monitoring and considering the rapid uptake of CGM, an international panel developed consensus recommendations for CGM-based glycemic targets,^7^ including the recommendation that women with pregestational T1D should spend >70% of time in range (63–140 mg/dL), <25% of time above range (>140 mg/dL), and <5% of time below range (<4% of time <63 mg/dL and <1% of time <54 mg/dL). These recommended targets were immediately endorsed by several professional societies, including the American Diabetes Association (ADA), the American Association of Diabetes Educators (AADE), the Foundation of European Nurses in Diabetes (FEND), the American Association of Clinical Endocrinologists (AACE), and the European Association for the Study of Diabetes (EASD).

Despite the recent international consensus statement and randomized controlled trial (RCT) evidence that CGM use is beneficial for pregnant women and their neonates, most CGM systems still do not include indications for use in pregnancy. This prospective observational study aimed at establishing the performance of the Dexcom G6 CGM System (G6) in pregnant women.

## Methods

This study was conducted at three investigational sites in the United States between May 13, 2019 and August 9, 2019 and included evaluation of the G6 CGM system (Dexcom, Inc., San Diego, CA) in women with diabetes in the second or third trimester of pregnancy (clinicaltrials.gov NCT03935191). Exclusion criteria for enrollees included the presence of extensive skin abnormalities at the sensor insertion site, a hematocrit value less than 30%, a clinical need for dialysis or tocolytic drugs, or condition(s) placing the participant at elevated risk for adverse maternal or neonatal outcomes. The study was approved by a central institutional review board, and written informed consent was obtained as required. Funding, supplies, and technical expertise were provided by Dexcom, Inc.

Each study participant wore two CGM devices on the abdomen, upper buttock, and/or posterior upper arm concurrently for up to 10 days. One was designated as the primary system and was blinded. The other system served as the secondary system and could be unblinded, to be used to inform treatment decisions. All systems placed on the posterior upper arm were blinded. The unblinded sensor location (abdomen or upper buttock) was determined by participant and investigator preference.

All participants completed one 6-h clinic session between days 3 and 7 of the sensor wear. During this time, both G6 systems were blinded and arterialized venous blood samples were collected at 30 ± 5 min intervals for measurement on the 2300 STAT Plus Glucose & Lactate Analyzer (YSI Life Sciences, Yellow Springs, OH). Meals and snacks were selected by study participants without restriction. Insulin and other diabetes medications were self-administered by participants per usual care throughout the clinic visit. Diabetes treatment decisions were based on SMBG. No protocol-specific manipulation of glucose levels to specific targets was performed. After the clinic session, the secondary Dexcom G6 CGM System was restored to the unblinded mode.

Each YSI value was paired with the CGM value that immediately followed and was within 5 min. Accuracy metrics included the proportion of the CGM system values that were within ±20% of paired YSI values >100 mg/dL or ±20 mg/dL of YSI values ≤100 mg/dL (hereafter referred to as %20/20), as well as the analogous %15/15, %30/30 and %40/40. The absolute difference (AD) and absolute relative difference (ARD) were calculated for each pair of values as |EGV_i_–YSI_i_| and |EGV_i_–YSI_i_| ÷ YSI_i_, respectively. Mean AD (MAD) and mean ARD (MARD) were calculated as means of sets of AD and ARD values, respectively. The MAD was calculated for any estimated glucose value (EGV)-YSI pair for which the YSI value was in level 2 (<54 mg/dL) or level 1 (<70 mg/dL) hypoglycemia; MARD was calculated for any EGV-YSI pair for which the YSI value was in level 1 (>180 mg/dL) or level 2 (>250 mg/dL) hypoglycemia. There was no pre-specified hypothesis, as this was an observational study. All analyses were performed by using SAS^®^ software, version 9.3 (SAS Institute, Inc., Cary, NC).

Safety was characterized by the incidence of device-related adverse events (AEs). Skin irritation at the sensor wire insertion site and at the area underlying the adhesive patch were evaluated after each sensor removal. Any edema and/or erythema observed at the sensor insertion site or adhesive area was evaluated according to the Draize's scale.^30^ All participants who underwent sensor insertion were included in the safety analysis.

## Results

### Study population

After screening, 32 pregnant women ages 18–41 years were enrolled. Demographic and baseline characteristics are summarized in Table 1. Fourteen (43.7%) participants self-identified as non-White. All 20 participants with T1D used insulin (15 via continuous subcutaneous insulin infusion and 5 via multiple daily injections [MDI]); 2 out of the 3 with T2D used insulin (both via MDI); and 4 out of the 9 with GDM used insulin (all via MDI).

**Table 1.** Participant Demographics and Baseline Characteristics

Characteristic	Participants (*n = *32)
Age, years	30 ± 5.9
Duration of diabetes, years	9.7 ± 9.2
Type of diabetes, *n* (%)	
Type 1	20 (62.5)
Type 2	3 (9.4)
Gestational	9 (28.1)
HbA1c, %	6.1 ± 1.2
Pregnancy trimester, *n* (%)	
Second	19 (59.4)
Third	13 (40.6)
CGM use in previous 6 months, *n* (%)	
Yes	18 (56.3)
No	14 (43.8)
Insulin use, *n* (%)	
Yes	26 (81.3)
No	6 (18.8)

Descriptive statistics are presented as mean ± standard deviation.

CGM, continuous glucose monitoring.

### Distribution of sensor wear sites

Sixty-eight sensors were applied, four of which were replacements for two participants. Of these sensors, 25 (36.8%) were inserted on the front or lateral abdomen, 26 (38.2%) were inserted on the posterior upper arm, and 17 (25.0%) were inserted on the buttock. The most frequent configuration was having one sensor on the abdomen and the other on the posterior upper arm (16 participants), followed by one sensor on the posterior upper arm and the other on the buttock (10 participants).

### Accuracy

All participants contributed at least one YSI-EGV matched pair data and were included in the analysis population. A total of 734 matched pairs were collected. The overall %20/20 accuracy was 92.5%, and site-specific %20/20 accuracies were 91.6%, 95.9%, and 87.7% for sensors worn on the abdomen, posterior upper arm, and buttock, respectively (Table 2). The overall %30/30 was 98.4%, indicating a few outliers. The overall MARD was 10.3% (11.5% for sensors worn on the abdomen, 8.7% for sensors worn on the posterior upper arm, and 11.2% for sensors worn on the buttock). The MAD in YSI-measured hypoglycemia between 54–69 mg/dL was 9.0 mg/dL and between 40–53 mg/dL was 6.4 mg/dL, indicating acceptable accuracy in hypoglycemic ranges (Table 3).

**Table 2.** Analytical Accuracy During Frequent Sample Testing, by Wear Site

	All wear sites (sensor* n* = 63*^a^*)	Sensor wear site		
		Abdomen (sensor* n* = 25)	Posterior upper arm (sensor* n* = 25)	Upper buttock (sensor* n* = 13)
Accuracy metric	Matched pairs = 734	Matched pairs = 285	Matched pairs = 294	Matched pairs = 155
MARD (%)	10.3	11.5	8.7	11.2
%15/15 (%)	81.1	77.9	86.1	77.4
%20/20 (%)	92.5	91.6	95.9	87.7
%30/30 (%)	98.4	97.9	99.7	96.8
%40/40 (%)	99.5	98.6	100.0	100.0

^a^ Sixty-eight sensors were applied; four were replacements, and one had an adhesive failure and did not contribute any matched pair data.

MARD, mean absolute relative difference.

**Table 3.** Percent and Point Accuracy Across Glucose Ranges

YSI glucose (mg/dL)	Matched pairs (*n*)	%15/15 (%)	%20/20 (%)	%30/30 (%)	MAD (mg/dL)	MARD (%)
40–53	8	100.0	100.0	100.0	6.4	—
54–69	24	79.2	91.7	100.0	9.0	—
70–180	674	80.7	92.4	98.5	—	10.2
181–250	28	85.7	92.9	92.9	—	7.9

MAD, mean absolute difference; MARD, mean absolute relative difference.

### Device-related AEs

There were no device-related AEs. There were three AEs unrelated to the device or study procedures. There were no moderate or severe insertion site reactions and no insertion site infections. Similarly, there were no moderate or severe adhesive reactions and no adhesive area infections or adhesive-related skin injuries. There were eight very slight erythematous reactions (two abdomen, three posterior upper arm, and three buttock) and eight very slight edematous reactions (one abdomen, two posterior upper arm, and five buttock) at the insertion sites. Pregnant women tolerated sensor wear well, regardless of wear site.

## Discussion

This study demonstrated that the Dexcom G6 CGM System provided accurate readings in comparison to reference YSI blood glucose values and that sensors were well tolerated and performed well whether placed on the abdomen, buttock, or posterior upper arm in pregnant women with diabetes. The overall accuracy metrics reported here (MARD = 10.3%, %20/20 = 92.5%) are comparable to accuracy metrics for the same system used in non-pregnant adults and inserted on the abdomen (MARD = 9.8%, %20/20 = 92.5%).^31^ Accuracy was best for sensors placed on the posterior upper arm. Participants remained recumbent throughout YSI testing, thus it is possible that positional pressure on some sensors may have contributed to the higher MARD for the abdomen (which include sensors inserted on the lateral abdomen) and buttocks. An earlier study of Dexcom G4 sensors^32^ found similar accuracies for sensors placed on the abdomen and arm, and a separate study of FreeStyle Libre sensors^33^ found that sensors placed on the abdomen were less accurate than those placed on the arm. The notion that CGM accuracy is not different in pregnant and non-pregnant populations is supported by the present data, as well as by data from earlier studies of the FreeStyle Navigator^34^ and Libre^35^ systems in pregnant women. Glucose monitoring device accuracy is particularly important in pregnancy, as the target glucose range is much narrower than for non-pregnant adults. Accordingly, it is important that pregnant women are willing and able to perform SMBG for the times that the CGM readings are discordant from symptoms or expectations. This is particularly important for women new to CGM and in the first day after sensors are inserted.

The G6 System was recently CE marked for use in pregnancy and for wear on the posterior upper arm. The previous label carried a warning against its use in pregnancy; removing the warning has the potential to reduce the high burden of diabetes management in pregnancy and may improve a woman's ability to obtain the device during pregnancy. The advantages of CGM over SMBG testing alone have been demonstrated in RCTs showing that CGM contributes to improved maternal and neonatal outcomes in T1D.^24,36^ The CGM may also be beneficial in women with other types of diabetes. A systematic review by Yu et al.^37^ reported that CGM was superior to SMBG in women with GDM for detecting hypoglycemic and hyperglycemic excursions, which may result in improved maternal and fetal outcomes for CGM users with GDM.

One limitation of this study is its small sample size, which prevents meaningful assessment of accuracy as a function of stage of pregnancy, type of diabetes, use of insulin, or sensor location. Since intentional glucose manipulations were not conducted, a second limitation is the paucity of reference glucose measurements in both level 1 and level 2 hypoglycemia or hyperglycemia ranges. Strengths of our study include the use of reference blood glucose values for comparison, inclusion of multiple sensor wear sites, and inclusion of participants with a variety of diabetes types during pregnancy. Additional studies of the influence of wear site on sensor performance are warranted, as are studies of glucose fluxes within the abdominal skin of pregnant women. Future studies in pregnant women with diabetes should evaluate how to best utilize customizable G6 features, such as data sharing, alerts, and summary reports that are specific to pregnancy, to further improve glycemic control.
